# Study of the value of homocysteine levels in predicting cognitive dysfunction in patients after acute carbon monoxide poisoning

**DOI:** 10.1186/s12873-022-00684-8

**Published:** 2022-07-19

**Authors:** Wei Ren, Xiao Shuai Zhou

**Affiliations:** 1 Emergency Department, Ningbo Yinzhou No. 2 Hospital, 998 Qianhe Road, Yinzhou, Ningbo, Zhejiang, China; 2 Ningbo Yinzhou No. 2 Hospital, 998 Qianhe Road, Yinzhou, Ningbo, Zhejiang, China

**Keywords:** Carbon monoxide poisoning, Cognitive dysfunction, Homocysteine, Hyperhomocysteinemia

## Abstract

**Purpose:**

The purpose of this research was to assess the value of homocysteine (HCY) levels in predicting cognitive dysfunction in patients after acute carbon monoxide (CO) poisoning.

**Methods:**

A total of 115 patients who were admitted to the emergency department of Yinzhou NO. 2 Hospital after CO poisoning between January 2017 and December 2021 were enrolled in this retrospective study. All patients were followed up for 1 month. According to the Mini–Mental State Examination (MMSE) scores, patients were divided into two groups. The demographic and clinical characteristics and magnetic resonance imaging (MRI) results were gathered and statistically analysed.

**Results:**

Twenty-six and 89 patients were ultimately enrolled in the cognitive dysfunction and control groups, respectively. There were significant differences between the groups in terms of age, coma duration, and carboxyhaemoglobin (COHB), lactate and HCY levels (*p* < 0.05), but there were no significant differences in white blood cell (WBC) counts or aspartate transaminase (AST), alanine transaminase (ALT), creatinine, troponin T, creatinine kinase (CK), or creatinine kinase muscle and brain (CK-MB) levels (*p* > 0.05). Univariate and multivariate analyses identified that a higher HCY level (OR 2.979, 95% CI 1.851-5.596, *p* < 0.001) was an independent risk factor for patient cognitive dysfunction after acute CO poisoning. Linear regression analysis showed a negative correlation between MMSE scores and HCY levels (*r* = − 0.880, *P* < 0.001). According to the MRI results, the most common lesion site was the globus pallidus, and the central ovale, diffuse white matter, corona radiata, basal ganglia (other than the globus pallidus) and cerebral cortex were also involved.

**Conclusions:**

Higher HCY levels were associated with cognitive impairment and were independent risk factors for cognitive impairment after acute CO poisoning. The level of HCY was negatively correlated with the degree of cognitive impairment.

## Introduction

Acute carbon monoxide poisoning (ACOP) is a common life poisoning and occupational poisoning in many countries and has high morbidity and mortality. Carbon monoxide (CO), which is odourless, colourless, and tasteless, is well known as a silent killer, as it binds with haemoglobin in the blood with almost 300 times higher affinity than oxygen, affects the O_2_-carrying ability of blood, and causes hypoxia in tissues [1]. In addition, CO can bind to ferrous iron in reduced cytochrome oxidases, which exaggerates hypoxia. The organs with the highest demand for oxygen, such as the brain and the heart, are more vulnerable to injury. Many studies have shown that CO poisoning can result in acute neurological sequelae and cognitive sequelae and delay neurological sequelae [2]. The main pathological changes are extensive demyelination of brain white matter, bilateral symmetrical pallidocyte ischaemia and necrosis [3–5].

Homocysteine is a sulfur-containing amino acid derived from methionine that is a product of normal folate and methionine metabolism. Elevated levels of homocysteine, a condition called hyperhomocysteinaemia (hHCY), have been linked to neurological diseases and an increased risk of cardiovascular diseases. Data from a study by Kovalska et al. [6] indicated that the combination of hHCY and ischaemia–reperfusion insult exacerbated hippocampal neurodegenerative processes in rats, demonstrating that hHCY represents a strong risk factor for atherosclerosis-associated diseases, such as stroke, dementia and Alzheimer’s disease. In a variety of clinical studies, elevated plasma HCY levels were independently associated with cognitive decline and dementia [7, 8]. Plasma HCY levels are associated with white matter lesions, hippocampal atrophy and lacunar infarcts in cross-sectional studies [5, 9]. However, there are few studies on the correlation between homocysteine and cognitive sequelae in patients with acute carbon monoxide poisoning.

In the present study, we mainly determined the relationship between plasma homocysteine levels and cognitive dysfunction in patients with CO poisoning, and we assessed the value of plasma HCY levels in predicting cognitive dysfunction in these patients.

## Materials and methods

### Study design

This retrospective cohort study adhered to the tenets of the Declaration of Helsinki. This study was approved by the Ethics Committee of Ningbo Yinzhou NO. 2 Hospital. Additionally, written informed consent was obtained from the patients.

### Patient selection

Patients who were hospitalized at Yinzhou NO. 2 Hospital, Ningbo, China, for acute CO poisoning from January 2017 to December 2021 were enrolled in this study. Acute CO poisoning was diagnosed on the basis of a history of CO exposure and symptoms of acute poisoning, with elevated arterial carboxyhaemoglobin (COHB) (COHB > 5% in nonsmokers and 10% in smokers). All patients were followed up for one month. Patients with any of the following conditions were excluded: 1) age < 15 years; 2) uncertain exposure history; 3) a history of neurological diseases, including dementia, psychiatric disease or Parkinson’s disease; and 4) missing data. Magnetic resonance imaging (MRI) was pivotal in the assessment of brain changes in patients after CO poisoning. All patients underwent blood examination and MRI at admission and received hyperbaric-oxygen therapy (HBOT) to reduce the possibility of cognitive sequelae. The Hcy level was detected by the cyclic enzymatic method. The QR-5200 model automatic biochemical analyzer was used to detect the biochemical indicators. Demographic data, physical examination findings, laboratory findings and imaging data were extracted from the electronic medical records of registry-enrolled patients. The Mini–Mental State Examination (MMSE) was used to assess the severity of cognitive impairment. The MMSE was administered 4 weeks after CO exposure. The MMSE consists of 30 questions with a maximum score of 30.

An MMSE score < 27, a cut-off point that differentiates normal cognition from mild cognitive impairment and dementia [10–12], was considered to indicate cognitive impairment.

### Statistical analysis

Data were analysed with Statistical Package for Social Sciences (SPSS) software, version 23.0 for Windows (IBM, USA). Data are presented as the mean ± standard deviation, median with interquartile range or frequency. Student’s t test and the chi-squared test were performed to investigate the difference between the two groups on demographic and clinical variables. Uni- and multivariate logistic regression were performed to analyse risk factors associated with cognitive dysfunction. Linear regression analysis was used to assess the correlation of MMSE scores and HCY levels. The confidence interval (CI) was determined as 95%, and a two-tailed *p* value less than 0.05 was considered to indicate a statistically significant result.

## Results

A total of 130 acute CO-poisoned patients were included in our study. Fifteen patients were excluded owing to a history of cerebrovascular disease or missing data. Therefore, the data of 115 patients were analysed. According to the MMSE scores, the patients were divided into two groups: the cognitive dysfunction group (*n* = 26) and the control group (*n* = 89). The patients’ characteristics in each group are shown in Table 1. The mean age of the cognitive dysfunction group was 47.53 ± 15.36 years, while the mean age of the control group was 37.09 ± 12.58 years, with the difference between groups being statistically significant (*p* = 0.001). Compared with the control group, coma duration (*p* = 0.001) and HCY level (*p* < 0.001) were significantly higher in the cognitive dysfunction group. There was no statistically significant difference between the two groups in terms of sex (*p* = 0.246) or blood pressure (*p* = 0.58, *p* = 0.59).

There were no significant differences between the two groups with respect to WBC count or AST, ALT, Cre, troponin T, CK or CK-MB levels (*p* > 0.05). A weak but still statistically significant (0.05 > *p* > 0.01) correlation between COHB and lactate levels was detected in the two groups.

**Table 1:** Baseline characteristics of the patient groups

Variable	Reference range	Cognitive dysfunction group (*n* = 26)	Control group (*n* = 89)	*P*
Age (years)		47.53 ± 15.36	37.09 ± 12.58	0.001
Male/female		11/14	29/60	0.246
SBP (mmHg)	< 140	138 ± 15	136 ± 18	0.580
DBP (mmHg)	< 90	87 ± 12	88 ± 10	0.590
COHB (%)	0-1.5	31.90 ± 15.51	24.99 ± 13.67	0.030
Lactate (mmol/L)	0.5-1.7	4.57 ± 3.44	3.05 ± 2.12	0.041
WBC count(*10^9^/L)	3.5-9.5	12.53 ± 6.24	10.12 ± 4.77	0.077
AST (U/L)	13-35	23.49 ± 23.25	17.29 ± 6.03	0.190
ALT (U/L)	7-40	20.05 ± 13.26	19.27 ± 10.25	0.751
Cre (μmol/L)	45-84	59.50 ± 12.12	58.05 ± 14.24	0.637
BUN (mmol/L)	1.43-7.14	5.73 ± 1.67	4.82 ± 1.35	0.005
Troponin T (ng/ml)	0.01-0.023	0.078 ± 0.18	0.016 ± 0.020	0.088
CK (U/L)	26-140	299.65 ± 652.56	126.87 ± 193.24	0.194
CK-MB (U/L)	0-25	12.53 ± 6.24	10.12 ± 4.77	0.300
HCY (μmol/L)	0-15	21.03 ± 2.97	13.73 ± 2.06	0.000

*Abbreviations*: *SBP* Systolic blood pressure, *DBP* Diastolic blood pressure, *WBC* White blood cell, *AST* Aspartate transaminase, *ALT* Alanine transaminase, *Cre* Creatinine, *BUN* Blood urea nitrogen, *CK* Creatinine kinase, *CK-MB* Creatinine kinase muscle and brain, *HCY* Homocysteine

**Fig. 1:**
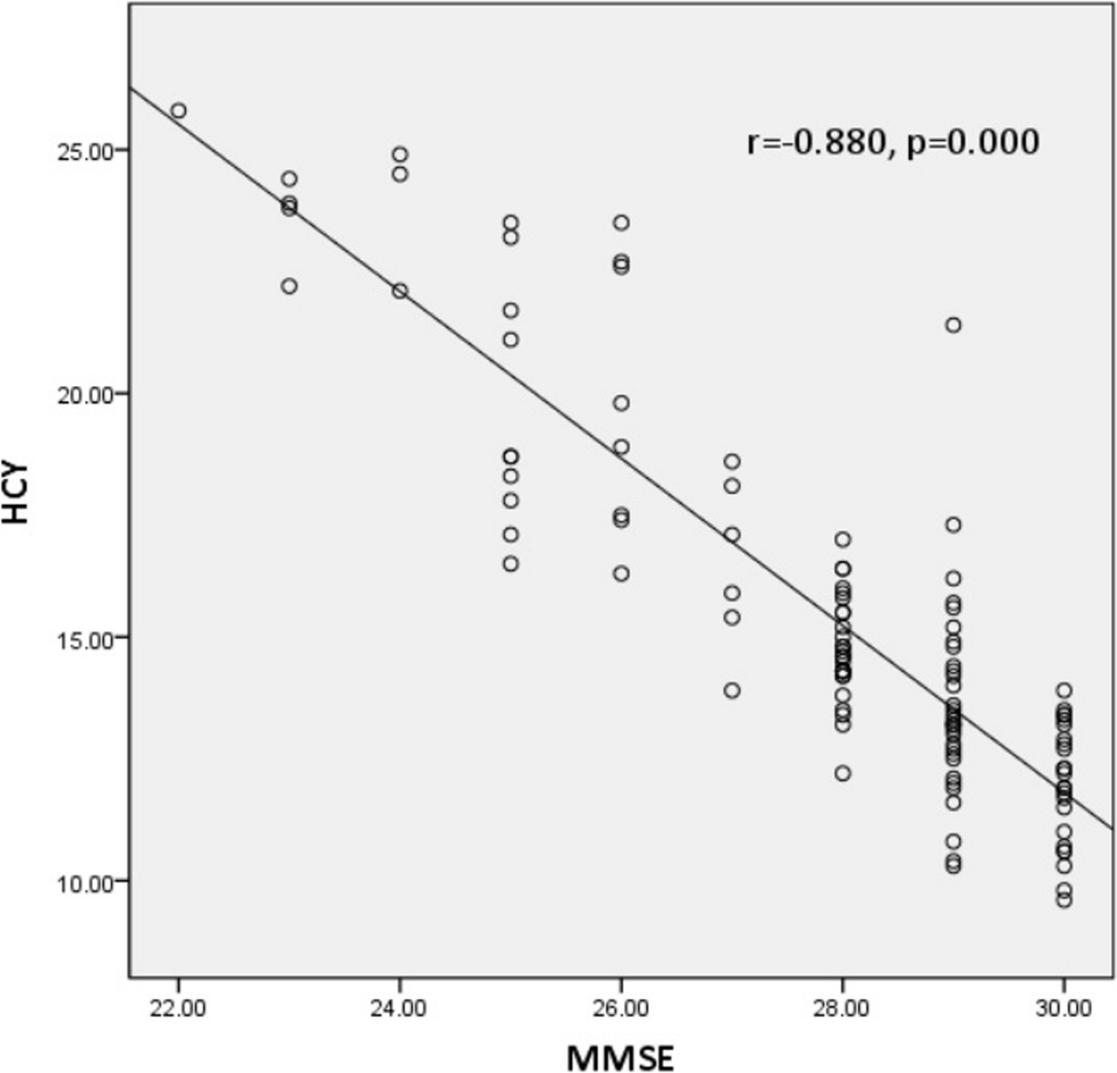
Homocysteine levels were negatively correlated with MMSE scores

In the study, we also found that advanced age (OR: 1.057, *p* = 0.002) and elevated COHB (OR: 1.035, *p* = 0.034), lactate (OR: 1.233, *p* = 0.011), WBC counts (OR = 1.084, *p* = 0.044), BUN (OR: 1.498, *p* = 0.008), and HCY (OR: 2.979, *p* < 0.001) were risk factors for cognitive dysfunction (Table 2). Our multivariate regression analysis showed that HCY was an independent factor associated with cognitive impairment in patients after CO poisoning (OR: 3.218, *p* < 0.01). Finally, we conducted correlation analyses between MMSE scores and HCY levels. As predicted, we found a negative correlation between MMSE scores and HCY levels (Fig. 1, *r* = − 0.880, *P* < 0.001).

**Table 2:** Predictors of cognitive dysfunction in patients with CO poisoning

Variable	Univariate analysis		Multivariate analysis	
	OR (95% CI)	*p*	OR(95% CI)	*p*
Age (years)	**1.057 (1.021-1.095)**	**0.002**	**1.005 (0.935-1.081)**	**0.883**
COHB (%)	**1.035 (1.003-1.069)**	**0.034**	**0.994 (0.891-1.109)**	**0.918**
Lactate (mmol/L)	**1.233 (1.049-1.451)**	**0.011**	**0.970 (0.470-2.003)**	**0.935**
WBC count (*10^9^/L)	**1.084 (1.002-1.172)**	**0.044**	**0.883 (0.693-1.126)**	**0.883**
AST (U/L)	1.045 (0.989-1.103)	0.116		
ALT (U/L)	1.006 (0.968-1.046)	0.749		
Cre (mg/dl)	1.008 (0.976-1.040)	0.634		
BUN (mg/dl)	**1.498 (1.112-2.019)**	**0.008**	**1.635 (0.783-5.569)**	**0.191**
CK (IU/L)	1.001 (1.000-1.002)	0.085		
CK-MB (IU/L)	1.010 (0.990-1.031)	0.328		
tHCY (μmol/L)	**2.979 (1.780-4.985)**	**< 0.001**	**3.218 (1.851-5.596)**	**< 0.001**

*Abbreviations*: *SBP* Systolic blood pressure, *DBP* Diastolic blood pressure, *WBC* White blood cell, *AST* Aspartate transaminase, *ALT* Alanine transaminase, *Cre* Creatinine, *BUN* Blood urea nitrogen, *CK* Creatinine kinase, *CK-MB* Creatinine kinase muscle and brain, *HCY* Homocysteine

All patients’ MRI results were analysed. Thirty out of 115 patients (26. 09%) had signal changes on MRI, including 4 patients in the control group and 26 patients in the cognitive dysfunction group (Table 3). Twelve patients had multiple lesions. Among these 12 patients, lesions in the corona radiata, centrum ovale and globus pallidus were observed in 4 patients. Lesions in the basal ganglia (other than the globus pallidus), centrum ovale, and paraventricular nucleus were observed in 3 patients. Lesions in the globus pallidus and centrum ovale were observed in 2 patients. Seven patients showed demyelinating lesions in the diffuse cerebral white matter. Lesions were observed in exclusively the globus pallidus in 6 patients, the frontal cortex in 3 patients, the cerebellum in one patient, and the hippocampus in one patient. In the cognitive dysfunction group, the most common lesion site was the globus pallidus (50%). The central ovale (38.46%), diffuse white matter (23.08%), corona radiata (15.38%), basal ganglia (other than the globus pallidus) (11.54%) and cerebral cortex (11.54%) were also involved.

**Table 3:** Lesion sites in patients with brain lesions on MRI

Lesion site	No. (%)		
	Total (*n* = 115)	Cognitive dysfunction group (*n* = 26)	Control group (*n* = 89)
Diffuse white matter	7	6 (23.08)	2 (2.24)
Globus pallidus	14	13 (50.00)	1 (1.12)
Basal ganglia other than the globus pallidus	4	3 (11.54)	2 (1.12)
Centrum ovale	12	10 (38.46)	2 (2.24)
Corona radiata	5	4 (15.38)	1 (1.12)
Cerebral cortex	3	3 (11.54)	0 (0)
Parietal lobe	0	0 (0)	0 (0)
Temporal lobe	1	1 (3.85)	0 (0)
Occipital lobe	0	0 (0)	0 (0)
Hippocampus	1	1 (3.85)	0 (0)
Corpus callosum	0	0 (0)	0 (0)
Paraventricular nucleus	4	1 (3.85)	1 (1.12)
Cerebellum	1	1 (3.85)	0 (0)

## Discussion

To the best of our knowledge, this study was the first to explore the relationship between plasma HCY levels and cognitive dysfunction in patients after CO poisoning. In our study, we found that the group with cognitive dysfunction had higher Hcy levels than the control group. Significant differences in age and plasma blood urea nitrogen levels were observed in the two groups. As shown in Table 2, we found that an elevated WBC count was related to cognitive dysfunction, mainly attributable to oxidative stress and inflammation [13]. However, whether anti-inflammatory therapy can prevent cognitive dysfunction is still unknown [14]. Previously published studies have reported that elevated HCY levels are a risk factor for several pathological disorders, such as acute stroke, Alzheimer’s disease, and Parkinson’s disease [15–18]. In the present study, high levels of HCY were clearly an independent risk factor for cognitive dysfunction. Figure 1 demonstrates the significant negative correlation of HCY levels with MMSE scores. Our results agree with those of Grzegorz Raszewski et al., who also showed a significant relationship between MMSE scores and serum levels of HCY in dementia [19].

An observational study indicated that the presence of acute brain lesions was independently associated with the development of delayed neurological sequelae after acute CO poisoning, and the globus pallidus was the most common lesion site [20, 21]. Moreover, other studies reported that the majority of the lesions in delayed encephalopathy after CO poisoning were located in the globus pallidus, subcortical white matter, and basal ganglia [22]. We observed similar findings in our study. The white matter of the cerebral hemispheres, especially in the globus pallidus, was involved in patients complicated with cognitive dysfunction. The cerebral cortex, hippocampus, and cerebellum were also affected. Extensive clinical studies support that the plasma HCY concentration is associated with hippocampal atrophy, white matter lesions and lacunar infarcts [5, 9]. This may explain the correlation between HCY and cognitive dysfunction after CO poisoning on imaging.

Existing studies have demonstrated that elevated HCY causes cytotoxicity and proinflammatory effects, leading to endothelial dysfunction, lipid metabolism disorders and vascular cognitive impairment [23, 24]. Oxidative stress, DNA damage, protein thiolation or protein homocysteinylation, triggering apoptosis and excitotoxicity, all contribute to the HCY-mediated pathomechanism of neurological disorders [25]. However, the mechanisms underlying HCY and cognitive dysfunction after acute CO poisoning need considerable additional research to be validated.

### Limitations

The first limitation of the study is the small sample size, with few patients. Second, in this study, all patients were followed up for 1 month, but some patients may have developed cognitive dysfunction after that time period. Third, only the MMSE was used to measure cognitive function, and other cognitive function assessments should be applied in future studies.

## Conclusion

In summary, our study investigated the value of homocysteine levels in predicting cognitive dysfunction in patients after acute CO poisoning. We found that there was a negative linear correlation between plasma HCY levels and MMSE scores. High HCY levels were associated with cognitive impairment and were independent risk factors for cognitive dysfunction. Further prospective studies are needed to assess whether early intervention to normalize HCY levels can prevent cognitive dysfunction in patients with acute CO poisoning.

## Data Availability

The datasets generated and/or analysed during the current study are not publicly available due to the privacy of the patients, but they are available from the corresponding author upon reasonable request.

## Abbreviations

ACOP: Acute carbon monoxide poisoning
CO: Carbon monoxide
COHB: Carboxyhaemoglobin
HCY: Homocysteine
hHCY: Hyperhomocysteinemia
SPSS: Statistical Package for Social Sciences
MMSE: Mini-Mental State Examination
MRI: Magnetic resonance imaging
